# Behaviours of farmed saltwater crocodiles (*Crocodylus porosus*) housed individually or in groups

**DOI:** 10.3389/fvets.2024.1394198

**Published:** 2024-07-08

**Authors:** Dana L. M. Campbell, Leisha Hewitt, Caroline Lee, Charlotte A. Timmerhues, Alison H. Small

**Affiliations:** ^1^Agriculture and Food, Commonwealth Scientific and Industrial Research Organisation (CSIRO), Armidale, NSW, Australia; ^2^Consultant, Franklin, TAS, Australia; ^3^School of Biological and Environmental Sciences, Liverpool John Moores University, Liverpool, United Kingdom

**Keywords:** crocodilian, choice, welfare, commercial farm, reptile, threat perception

## Abstract

Saltwater crocodiles (*Crocodylus porosus*) are farmed in Australia primarily for their skins and meat. Commercially, they are raised in group pens as hatchlings and grower crocodiles and then moved to unitised (individual) pens for the final finishing stage when they are several years old. They will exhibit aggressive behaviour towards each other in captivity. Unitised pens can prevent animal injury and teeth marks on the skins but may result in other social restrictions. Research into behavioural housing preferences could assist the industry and inform the process of guideline development for optimal crocodile management and welfare. This study assessed the impacts of two housing systems, unitised or group pens, in 20 commercial finishing crocodiles through measuring behavioural profiles of individuals from video recordings, including housing preference when given a choice. Both pens included water and an above-water shelf, but the crocodiles in unitised pens could also access underneath the shelf. A threat perception test was applied to assess anxiety when housed individually or in groups. However, it was difficult to apply a standardised stimulus to all animals that reliably elicited a behavioural response. Further work would be needed to validate this test for commercial reptiles as the outcomes were not robust. The behavioural observation results showed clear differences in where the crocodiles spent their time across the day and in their activity levels between the pen types. However, interpretation of this variation was confounded by the physical and social differences between the pen types given the inconsistency in shelf access. Behaviours exhibited also differed given there were social opportunities in the group pens where individuals were observed engaged in both aggressive and non-aggressive contact interactions. In the free choice environment, crocodiles spent similar amounts of time in both unitised and group pens, suggesting there were features of both pen types that were attractive to the animals. However, skins were damaged from teeth marks highlighting the physical and economical risks of group housing. Further work could validate behavioural tests to quantify affective state impacts in different housing environments and whether social interactions do provide benefits for improving crocodile welfare.

## 1 Introduction

Saltwater crocodiles (*Crocodylus porosus*) are found in several regions of the southern and lower northern hemisphere including Australia and are farmed primarily for their skins and meat (1). Crocodile eggs are predominantly wild-harvested and are hatched on farm, where optimised conditions (2, 3) are essential for maximising hatchling survival and skin quality and thus the economic viability of the commercial industry (1, 4). Similar to societal pressures faced by other farmed livestock, the welfare of farmed crocodilians is a growing concern, with increasing pressure on the global industry to optimise animal welfare and develop standards and guidelines for management (5). However, there is as yet very little understanding of the behavioural ecology of captive saltwater crocodiles including what resources they desire or require under captive management.

Saltwater crocodiles are the largest species within the crocodile family (Crocodylidae) and considered to be the most aggressive (6, 7). In the wild, *C. porosus* hatchlings have been observed to remain together and with the (female) adult up to 2 months following hatching (8). Adult individuals occupy home ranges and will travel significant distances to return to these areas following human-mediated translocation indicating high site fidelity (9). However, individual tracking shows there is overlap in the areas that adult males will occupy and travel behaviour within home ranges varies across seasons and between males and females (10–12). In general, there are few published studies characterising the behaviour of saltwater crocodiles, either free-living or captive. Brien et al. (13) showed that aggressive (contact) behaviour between *C. porosus* individuals raised in captivity was present from 1 week of age and aggressive interactions were used to establish a dominance hierarchy as the hatchlings aged. The aggressive interactions grew in frequency in older hatchlings, but the proportion of interactions where both individuals were aggressive decreased significantly, indicating submission in the interactions (6, 13). Hatchling *C. porosus* observed at 13 and 40 weeks of age subject to aggression in captive conditions have been shown to flee to avoid the interaction (13). Observations of seven different crocodilian species in captivity showed that juvenile *C. porosus* at 12–18 months of age were rarely observed in close contact with each other compared with four of the observed species that would lie closely together in the water (6). More dominant individuals have been observed trying to exclude subordinate individuals from preferred areas of enclosures (13). Lower stocking densities in *C. porosus* hatchlings have been shown to increase the frequency of aggressive interactions (2). In the lower densities, one dominant individual was able to grow large at the expense of the others compared with higher densities where it appeared more individuals in close proximity prevented sustained aggressive interactions (2). Comparison between a breeding and non-breeding pond for adult *C. porosus* showed more aggression in the breeding pond which was also stocked at a lower stocking density (14). In commercial settings with grower Nile crocodiles (*Crocodylus niloticus*), higher stocking densities in group housing resulted in more skin teeth marks and more skin severely damaged from aggressive interactions (15). Olsson and Phalen (16, 17) reported that behaviour of saltwater crocodiles ‘returned to normal’ within 3–7 days post-sedation, but there was no characterisation of the pre-sedation behaviour nor were there descriptions of what constituted ‘normal behaviour’ in those studies.

Commercially in Australia, *C. porosus* are reared in both group and individual (unitised) pens (1) depending on the life stage. Group pens are used from hatching through ‘grow-out’ which can be several years; then, the animals are placed into the unitised (single) pens for the ‘finishing’ stage once the animals reach a certain body size (1). This finishing stage in unitised pens allows for healing of previous skin injuries, additional growth, and protection from aggressive interactions that can cause damage to and devalue the skin. However, some societal expectations dictate that farmed animals should be allowed social interaction facilitating display of appropriate and positive social behaviours, questioning the use of unitised pens. Dawkins (18) defined good welfare as animals being healthy and having what they want. With regard to farmed crocodiles, there is little information on what they ‘want’ as research in commercial settings is limited. It is unclear whether farmed crocodiles would benefit from social interactions with others and whether group housing during the finishing stage would facilitate positive affiliative or negative aggressive behaviours (5). Furthermore, there is limited research on whether farmed crocodiles require additional mental stimulation beyond feeding such as that provided through social interaction (7, 19), or the agency of environmental choice between land and water, without social competition. Social play by crocodilians is almost never reported—occasional reports of interactions between juveniles that may or may not be playful fighting or courtship behaviour do exist, but it is difficult to ascertain whether these are affiliative behaviours (20). Previous research on commercial Australian *C. porosus* farms comparing the use of unitised pens with group pens found no differences in plasma corticosterone concentrations between harvest-size individuals from unitised or group pens indicating neither housing type caused differences in stress responses (21). However, additional research into behavioural aspects would further determine how these two housing types impact on crocodiles’ welfare. There is a lack of evidence-based behavioural metrics available to assess welfare in captive reptiles (22) despite scientific literature indicating sentience and thus emotional capacity in reptilian species (23). Research into behavioural housing preferences could assist the industry to be better informed of good evidence-based farming practices and inform the process of policy, standards, and guideline development for optimal crocodile management.

The objective of the study was to assess the behavioural profiles of finishing crocodiles housed in unitised or group pens on a commercial farm. Behavioural preferences when given a choice of housing system were also measured as well as skin quality that can be impacted by social aggression. It was predicted that group housing would result in aggressive interactions and that the crocodiles would prefer the unitised pens. This study seeks to fill gaps in research surrounding the housing preferences of farmed saltwater crocodiles to inform commercial management guidelines that may optimise crocodile welfare.

## 2 Materials and methods

### 2.1 Ethical statement

This study was conducted under the authority of the CSIRO Wildlife and Large Animal, Animal Ethics Committee, reference 2020–20, in accordance with the Australian Code for the Care and Use of Animals for Scientific Purposes (24).

### 2.2 Animals and housing

A total of 20 crocodiles (*C. porosus*, *n* = 5 females, *n* = 15 males, 1.5–1.9 m in length, mean ± SEM 16.75 ± 0.59 kg body weight, approximately 3.5 years of age) were used for the study conducted between January and October 2021. Crocodiles were raised under commercial farm conditions in Queensland, Australia. They were raised in a group pen from hatching to approximately 34 weeks until transfer into group grower pens for at least 24 months. The animals were then moved to unitised (single) pens for the finishing period. In these pens, individuals were visually isolated via opaque pen walls, but they shared a body of water, circulated through gaps at the base of the dividing walls, and could hear and smell one another. The animals were pre-selected for the study at approximately 3 years of age and housed adjacent to one another in a single bank of 20 unitised pens for at least 4 months prior to the start of the study. The animals were reported to be healthy and ate well. Animals were fed three times per week, each animal receiving three chicken heads sourced from a local abattoir, dusted with a vitamin powder, at each feeding event.

### 2.3 Test arena facility design

**Figure 1:**
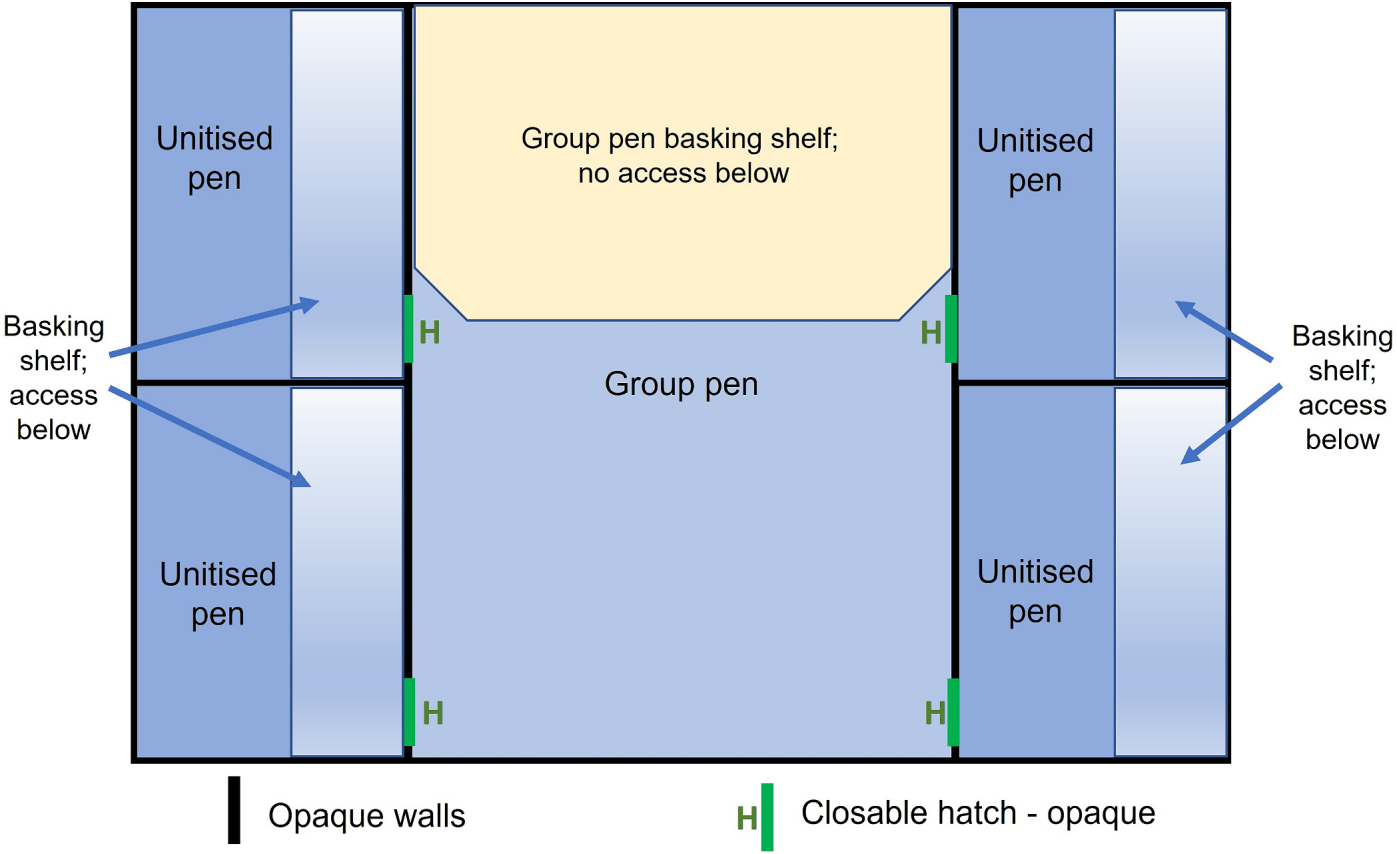
Schematic of the test arena facility housing four crocodiles showing the unitised (single) pens and the group pen. Basking shelves are indicated in the unitised pens which included access below the shelf. This underneath access was blocked in the group pen. Schematic is not drawn precisely to scale.

For the purposes of the study, a test facility was constructed of opaque PVC Celuka cellular foam measuring 12 mm thick, consisting of four unitised pens surrounding a central group pen area (Figure 1). Each unitised pen replicated a commercial unitised pen, being approximately 1 m wide and 2 m long. The pen contained a basking shelf (0.3 m wide × 2 m long) that ran along the length of the pen and the crocodile could lie on the shelf (out of the water) or access the area below the shelf (in water). Below the basking shelf, the base of the pen was filled with temperature-controlled water, which circulated through all the pens to a depth of 300 mm. The central group pen, designed to house four animals at a stocking density of 2 m^2^ per animal, provided a body of water (300 mm depth, approximately 70% of the total area of the pen) and a dry basking area approximately 2.4 m^2^ (approximately 30% of the total available area in the pen). There was no under-shelf area available in the group pen, which aligns with many commercial grower pens. To facilitate observation of animals and management, screened areas under which animals could lie were not available. As the potential aggression in the group pen was unknown in the current experiment, the decision was made to have no shelves to avoid an injured animal being trapped and hidden from view. All pen walls were constructed of opaque PVC Celuka cellular foam, so that there was no visual contact between pens, but the pens had a shared body of water and crocodiles were in auditory and scent contact.

A High-Definition Closed-Circuit Television (HDCCTV) system was installed to monitor behaviour of the animals. Two digital video cameras (EXIR Fixed Turret Network Camera, Model DS = 2CD2385G1-I, Hikvision, Hangzhou, China) were located above the test arena facility, such that each camera captured the entire floor area of two unitised pens and almost the entire floor area of the group pen. Continuous 24 h digital video footage was collected on a Network Video Recorder (Model DS-7608NI-I2/8P, Hikvision, Hangzhou, China). As an indication of climatic conditions in the test arena facility, temperature data were collected via two TinyTag^®^ (TG-4100 Aquatic 2, Hastings Data Loggers, Port Macquarie, Australia) loggers that recorded every 20 min throughout the day from in the water and from the ambient air in the period 12/01/2021 until 11/03/2021. Data were only available for this period of time due to data download failures.

### 2.4 Initial pilot study for threat perception testing

A threat perception test can be used as a measure of affective state where more anxious animals will have a heightened response to a perceived threat (25). To date, there have been no such tests validated on crocodilians. To identify an appropriate ‘threat’ to use, crocodiles were exposed to a novel stimulus in a pilot study. This pilot study was conducted in the bank of 20 unitised pens, prior to animals being moved to the test arena facility. Three potential stimuli were pilot-tested:

- An object was splashed into the water twice. This object was a 15 cm diameter rubber ring dog toy (Leaps & Bounds brand, PetBarn Pty Ltd., Chatswood, NSW, Australia) tied to a rope.
- Pressure was applied to the hind leg (thigh muscle) with the rounded end of a broom handle.
- The crocodile was tapped on a hind foot with the rounded end of a broom handle.

The behavioural response to each stimulus was assessed in real time and from HDCCTV video footage. One group of four pens was used per novel stimulus tested, such that each crocodile only experienced one putative ‘threat’ (12 of the 20 crocodiles experienced a ‘pilot’ threat). The hind leg pressure was observed to elicit the greatest response and thus was selected for use in the main study.

### 2.5 Main study

A total of five sequential replicates (numbered 1–5) of four animals each were used in the main study with the first replicate commencing in January 2021 and the final replicate finishing in October 2021. For each replicate, an individual crocodile was captured using electrical stunning methods (26–28), scutes were marked for identification while unconscious, and the animal was moved to a unitised pen in the test arena facility. Body weight and length measurements (snout tip to tail tip) were recorded prior to entry into the test arena and again on exit from the test arena facility. The degree of belly skin marks was scored upon entry and exit on a three-point scale by experienced commercial farm staff. In this scoring system, 1 was negligible visible marks, 2 was one or two light marks, and 3 equated to more than two light marks or more than one heavy mark that rendered the skin unsuitable for commercial sale. The marks graded were predominantly scars and open cuts. Light marks were those that only marked the surface or epidermal layers whereas heavy marks were deeper into the subdermal layers.

Once transferred to the test arena, the crocodiles were allowed 2 weeks to acclimate to the new location and recover from any stress associated with capture and handling before data collection began. Each replicate included behavioural recording in three phases: unitised pens (days 0–12), group pens where crocodiles no longer had access to the unitised pens (days 12–25), and free choice between both group and unitised pens (days 26–38). In the free choice, animals could enter any pen so there was a potential that multiple animals could enter the same pen. A threat perception test was applied on day 8 (animals in the unitised pens) and day 20 (animals in the group pens). This timeline is approximate as the timing of events such as the threat perception test and transfer into the group pen were adjusted to fit the workflow of the commercial farm, allowing for the availability of appropriate farm personnel.

For the threat perception test in the unitised pens, a single farm staff member applied the threat stimulus to each crocodile in the morning. The research team had previously instructed the member of staff on how to consistently administer the pressure with the broom to ensure uniformity in application across all crocodiles. It was not always possible to observe the crocodiles in the unitised pens as they were sometimes underneath the shelf at the time of testing. In such cases, the staff member applied the pressure to the hind leg based on their best visibility from above the pen. The reactions to the stimulus were video-recorded for later analysis. In the group pens, initially an object (baseball cap) was waved over the group by a single farm staff member as a threat stimulus able to be presented simultaneously to and visually perceived by the group given there was no shelf to hide under. However, this elicited almost no visible reaction. Thus, the pressure was applied to one hind leg of each individual in the group pen using a blunt broom handle, as had been applied to the individuals when housed in the unitised pens. This occurred 2 days after the object was waved over the pen for both replicates one and two, then 8 days after entering the group pen for the remaining replicates once it was decided the cap wave was not a suitable stimulus. These tests were conducted across all replicates, but due to video recording failures (associated with power outages during storm season in tropical Queensland), only the tests for replicates one, four, and five (unitised pens) and one, two, and four (group pens) were observed.

Throughout the trial period, faeces were collected when available from the facility, using a long-handled scoop. These were processed for metabolome and microbiome analyses, which will be reported on in a companion study.

### 2.6 Ethogram development and behavioural observations

To quantify how the crocodiles behaved across the day in the unitised and group pen environments, including when they had free choice between the two, ethograms specific to crocodiles in this commercial setting were developed. A single research technician made notes on behaviours observed (location, active or inactive, physical posture, and counts of specific behaviours) over a continuous 24 h block of footage for two crocodiles in unitised pens and four crocodiles in the group pen. These free-form notes were then compared against a catalogue of behaviours that had been described in the published literature (6, 7, 13, 29), and a draft ethogram was developed by the research team. The final ethograms for unitised and group pens are displayed in Table 1. Some behaviours differed between the two pen types given the animals could access underneath the shelf in the unitised pens only and could interact with other crocodiles in the group pens only.

**Table 1:** Ethogram for unitised and group pens showing the locations (including during free choice), postures, and behaviours recorded by either scan sampling or continuous observations.

Descriptor	Definition	
Method: scan sampling every 15 min for 24 h		
*Location–unitised pens*		
Under shelf	Majority of body is not visible	Combined for analysis as “In water”
In water whole body	Body fully visible in the water	
In water partially front	Head and front legs visible in the water, rear under shelf	
In water partially back	Back legs and tail visible in the water, front under shelf	
On shelf fully	Majority of body is on the shelf	Combined for analysis as “On shelf”
On shelf partially	Approximately 50% of the length of the animal is on the shelf, such that the forelegs are on the shelf and the hind legs in the water, or vice versa	
*Location (count number of animals in each location)–group pens*		
Tail in water	Tail submerged under water, front body on shelf	Combined for analysis as “In water”
Front in water	Front body submerged under water, tail on shelf	
Close to the shelf	In the water but tucked alongside the shelf	
In water whole body	Body fully visible in the water	
On shelf fully–separate	Majority of body is on the shelf, not in contact with another animal	Combined for analysis as “On shelf”
On shelf fully–touching	Majority of body is on the shelf, animals are in close contact	
On shelf partially	Approximately 50% of the length of the animal is on the shelf, such that the forelegs are on the shelf and the hind legs in the water, or vice versa	
*Active/inactive (unitised and group pens)*		
Active	Moving	
Inactive	Stationary	
*Posture (unitised and group pens)*		
Submerged	Fully under water	
Head out	Most of the head above water surface, body and tail submerged	
Nose out	Head tilted with snout above water surface, remainder of head submerged, body and tail submerged	
Nostrils and eyes out	Floating with nostrils and eyes above water surface, remainder of head submerged, body and tail submerged	
Head and tail raised	Head and tail above water surface, body submerged	
Method: Continuous 5 min block every hour for 24 h		
*Active behaviour (counts, can be once only event or a bout)*		
Feed manipulation or eating	Snapping at feed with mouth movements, investigating feed hatch (bout)	
Scratching	Itching with claws at the body or tail with one back leg (bout)	
Slap tail against the wall	Tail hit with contact against the wall (bout)	Combined for results as “Tail movement”
Tail movement	Tail shifting from side to side (bout)	
Moving backwards	Movement in a backwards motion (event)	
Nudging objects	Pushing feed with nose in water, no mouth movements (bout)	
Defecating	Time of defecation (event)	
Jumping	Attempts to climb the wall (event)	
Snapping at water/blowing bubbles	Jaw rapidly opening and closing with the mouth at the surface of the water, or submerging the nostrils and producing bubbles (bout)	
*Active behaviour (group pens only)*		
Tail wagging with speed	Tail moving vigorously from side to side (bout)	
Head slap	One animal swings their head sideways towards the other animal, keeping their jaws closed, could result in contact or not (event)	
Bites	Lunging at the other animal with jaws open, then closing (or attempting to close) the jaws onto the other animal (event)	
Aggressive interaction	Lunging or snapping at another crocodile (event)	
Number of displacements	Count how many animals moved during an aggressive interaction (event)	
Under/over	Animals climb onto one another or push under one another while on the shelf or in the water (event)	
Resting on one another	Animals not moving but lying with part of the body resting on another animal (event)	
*Location (count number of animals in each location during free choice every 15 min for 24h)*		
On shelf (in unitised pen)	All or the majority of the body is in contact with the top of the shelf	
Under shelf (in unitised pen)	Head and more than half the body is located beneath the shelf	
Visible in water (unitised pen)	At least head and more than half the body is in the water in the unitised pen	
On shelf (in group pen)	All or the majority of the body is in contact with the top of the shelf	
Visible in water (group pen)	At least head and more than half the body is in the water in the group pen	
Part in, part out	At the unitised and group pen entrance, body is halfway between both pen types	

At each scan sample observation, location was first recorded, then whether the animal was active or inactive, and finally the posture of the animal. Crocodiles could not go under the shelf in the group pens.

After the ethograms were developed, the observation methods involved: (1) scan sampling to note the location of the individual animal, whether it was active or inactive, and the physical posture it displayed while in the water, and (2) counts of specific behaviours occurring as single events or bouts over a continuous 5 min period. (1) The scan sampling was conducted every 15 min over a 24 h period across 4 days per unitised pen for each of the five replicates (summed total of 20 days observed) and every 15 min over a 24 h period across a summed total of 28 days for the group pens for all five replicates. The observation days were selected to occur before and after the threat perception test to account for any effect of the test (i.e., a stressful event) on typical behaviours exhibited. This resulted in variation in number of observation days for the group pens as the first two replicates had two threat perception tests conducted before a single stimulus was selected for the remaining replicates and video recording partially failed in replicate 3. Thus, total observation days for the group pens were as follows: replicates one and two = 7 days each; replicate three = 4 days; replicates four and five = 5 days each. (2) The counts of specific behaviours (as per the ethogram developed previously in Table 1) occurred over a continuous 5 min period every hour, starting on the hour, or within 2 min before or after the hour if the 5 min block was divided into parts as a result of the video capture equipment. The aim was that the observer could watch a 5 min continuous block without switching video files. The counts of behaviours were made on the same 24 h period over the same days as per the scan sampling. Behavioural counts were summed within the continuous 5 min observation period across all instances per individual in the unitised pens and across all instances for all individuals in the group pens. This observation protocol was validated by checking that the protocol generated data representative of the pattern of location and activity level that had been observed by watching 24 h blocks. Two observers trained together to ensure they were in agreement prior to decoding the video, one then decoded the unitised pen files, and the other decoded the group pen files. When either observer encountered a behaviour or posture that was difficult to clearly assign to a descriptor on the ethogram, the observers consulted one another to agree on the final selection.

For the free-choice phase between the unitised and group pens, observations of locations based on the ethogram in Table 1 were made by scan sampling every 15 min across 24 h periods for a summed total of 46 days. These observations were conducted by a single trained observer across varying days per replicate based on the number of video recordings available (replicate 1: 11 days; replicate 2: 14 days; replicate 3: 10 days; replicate 4: 9 days; replicate 5: 2 days).

The threat perception video across three replicates of single pens (*n* = 12 crocodiles) and three replicates of group pens (*n* = 12 crocodiles) was analysed by a single research technician based on the ethogram described in Table 2. The ethogram was developed by two behavioural researchers based on initial observations of the crocodiles’ reactions across a sample of tests. The location of the crocodile at the time of the test was recorded (under the shelf (unitised pens only), on the shelf, or in the water) and the immediate behavioural reaction to each separate administration of pressure to the thigh. The total number of times the pressure was applied per crocodile was also counted given this was (unexpectedly) observed to vary across individuals.

**Table 2:** Ethogram of the behaviours observed in crocodiles following pressure applied to the thigh during the threat perception test in unitised and group pens.

Descriptor	Definition
Not visible	Not visible, if you cannot see the reaction of the croc adequately because it is under the shelf (relevant to unitised pens only where the crocodiles could hide under the shelf)
No reaction	No discernible reaction to the applied pressure
Lunge snap	Body lunges forward at the broom and snaps with jaws (may or may not grab onto the broom handle)
Lunge no snap	Body lunges forward at the broom, does not snap with jaws
Tail movement	Tail swishes back and forth
Body moves away	Crocodile swims/walks away with their whole body
Foot away	Crocodile moves their foot/feet away (into their body)
Turn and snap	Crocodile turns to the broom and snaps with jaws but is not lunging forward
Turn	Crocodile turns
Turn/lunges and snaps	Crocodile turns/lunges at broom handle and snaps with jaws

### 2.7 Data and statistical analyses

#### 2.7.1 Temperature data

Temperature data recorded every 20 min throughout the day from a sensor in the water and from a sensor in the ambient air were compiled from 12/01/2021 until 11/03/2021. This dataset incorporated some of the days on which observations occurred for some replicates but also included many days when direct observations were not carried out. These data were plotted to visually display the typical variation in temperature across the day for the crocodiles.

#### 2.7.2 Behavioural data

Video decoding of unitised and group pens was compiled into datasets of the scan sampling and continuous behavioural observations (as per ethograms in Table 1). In total, the unitised pen scan sampling observations comprised 7,680 datapoints (96 daily scan points × 4 days × 4 pens × 5 replicates), and the unitised pen continuous behavioural observations comprised 1,920 datapoints (24 daily observation points × 4 days × 4 pens × 5 replicates). The group pen scan sampling observations comprised 2,449 datapoints (96 daily scan points × 28 days (summed across all 5 replicates as number of days varied between replicates) minus 239 points where video was missing due to technical failures). The group pen continuous behavioural observations comprised 672 datapoints (24 daily observations points × 28 days).

The 15 min scan sampling time points for both the unitised and group pens were divided into six time periods throughout the 24 h daily scan period (00:15–04:00, 04:15–08:00, 08:15–12:00, 12:15–16:00, 16:15–20:00, 20:15–00:00). To enable statistical comparisons between the unitised and group pens for variables of location (in water or on shelf) and activity (active or inactive) assessed in the scan sampling observations, the unitised pen data were summed across the four pens (to match the *n* = 4 crocodiles per group pen) to produce a dataset of 1,920 datapoints (96 daily scan points × 4 days × 5 replicates).

Using JMP® 16.1.0 (SAS Institute, Cary, NC, USA) with significance set at *p* < 0.05, the scan sampling observations of crocodile location were sinh–arcsinh-transformed (best fit transformation for the dataset), and the location of ‘in water’ was analysed using a general linear mixed model (GLMM) with ‘pen type,’ ‘time period,’ and their interaction as fixed effects and ‘day,’ ‘replicate,’ and ‘time nested within time period’ as random effects (the location of ‘on shelf’ was the corresponding opposite and so was not analysed). A separate GLMM with the same fixed and random effects was also applied to the sinh–arcsinh-transformed numbers of crocodiles observed as ‘active’ (the corresponding opposite of ‘inactive’ was not analysed). Restricted maximum likelihood estimation methods were applied. The mean number of crocodiles observed in different postures while in the water is presented in the results but was not statistically analysed.

The raw behavioural data from the continuous observations for the unitised pens (*n* = 1920/behaviour; 24 daily observation points × 4 days × 4 pens × 5 replicates) and group pens (*n* = 672/behaviour; 24 daily observation points × 28 days) are presented graphically to show the mean number of occurrences for each behaviour. The data were summed across all five replicates as the number of days varied between replicates. Some behavioural categories were combined as detailed in the ethograms (Table 1). Additional behaviours are presented for the group pens as animals were able to interact with each other.

The observations of locations (based on the ethogram in Table 1) every 15 min across 24 h periods during the unitised/group pen free choice testing were compiled (*n* = 3,849; 96 daily observations points × 46 days across 5 replicates; minus 567 missing points due to video recording failure). However, of the 3,849 datapoints, there were 336 in which one camera failed to record (across different replicates) meaning only the crocodiles visible on the remaining camera could be classified; this resulted in only 1, 2, or 3 crocodiles being identified of the 4 total. These datapoints were removed leaving a total of 3,513 datapoints to be analysed. To enable statistical comparisons between the different free choice locations, the data were averaged per six time periods (00:15–04:00, 04:15–08:00, 08:15–12:00, 12:15–16:00, 16:15–20:00, 20:15–00:00) per day per replicate (final dataset *n* = 243 per six possible locations). These values were sinh–arcsinh-transformed and assessed via a GLMM with ‘location’ and the interaction with ‘time period’ as fixed effects and ‘day’ and ‘replicate’ as random effects. Restricted maximum likelihood estimation methods were applied. Significance was set at a *p*-value of < 0.05 with *post-hoc* Tukey’s HSD tests applied where significant differences were present.

The observational data of crocodile reactions from the threat perception test were compiled across the initial stimulus testing and then the three replicates for the unitised pens and three replicates for the group pens. These results are presented descriptively only as this was a first application of a threat perception test in crocodiles and several test days missed being recorded. The body weight and skin scoring data comparing entry and exit measurements are also presented descriptively.

## 3 Results

### 3.1 Temperature

**Figure 2:**
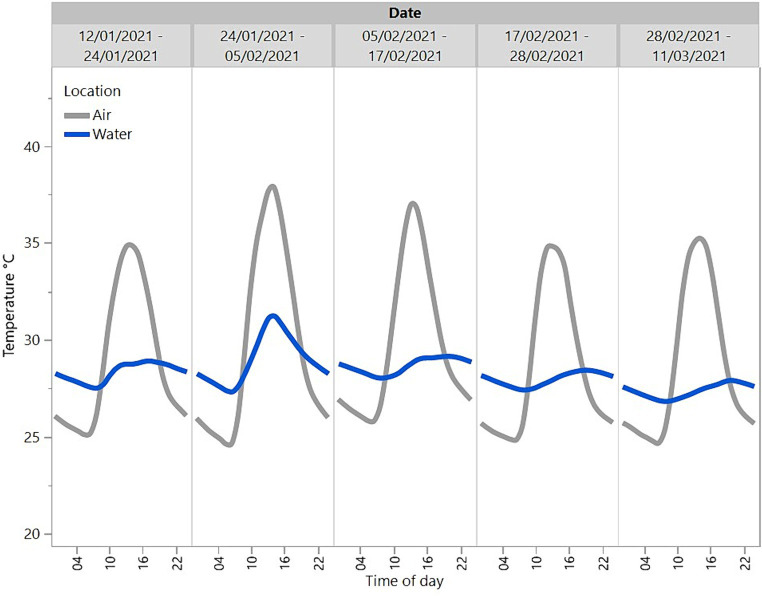
Mean temperatures across the day (24 h) in the ambient air and water of a sample crocodile pen across a period from 12/01/2021 to 11/03/2021.

Overall, the temperature of the water was more consistent across the day and cooler than the temperature in the ambient air across the summer months of January and February and part of autumn in March (Figure 2). The temperature showed a diurnal pattern and peaked around midday with the water warmer than the ambient air overnight.

### 3.2 Location of crocodiles

**Figure 3:**
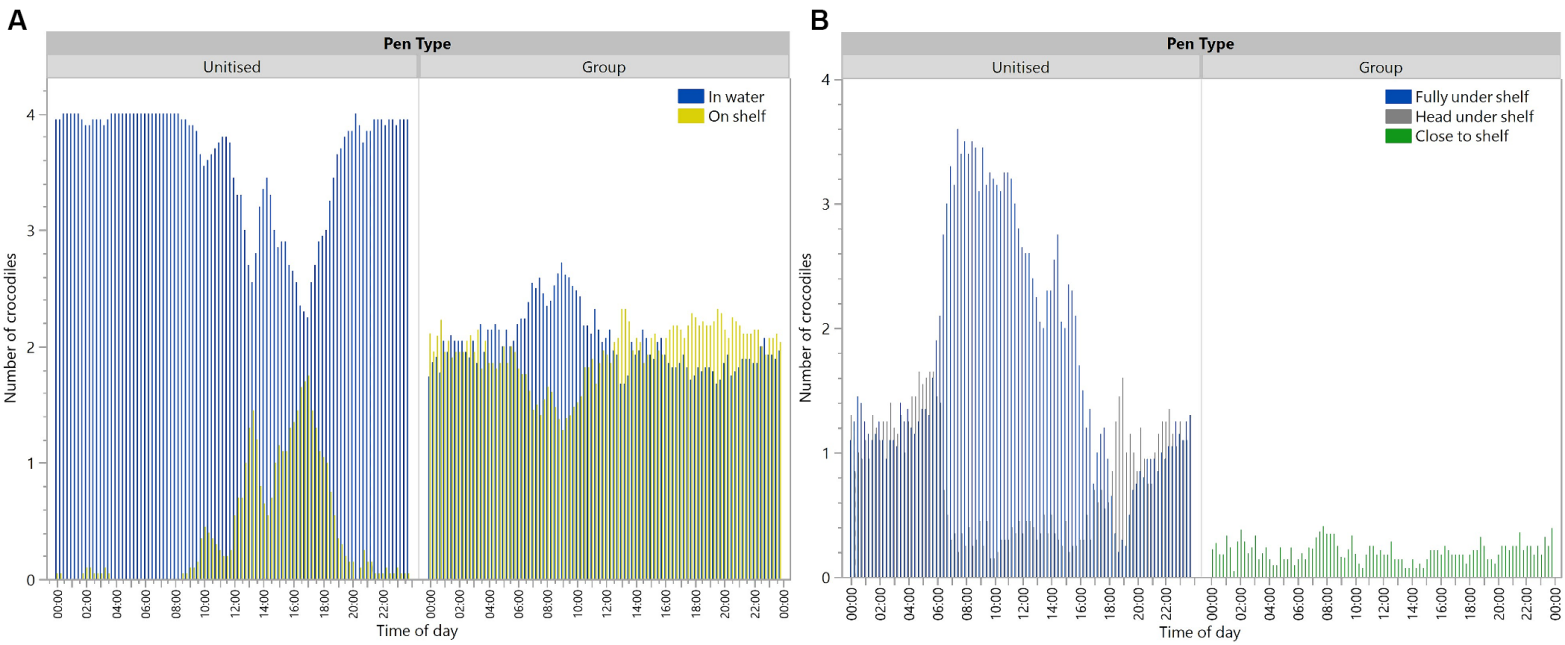
Number of crocodiles in unitised or group pens that were in the water or on the shelf **(A)**, or under/close to the shelf **(B)** at each 15 min scan observation point across the day. Crocodiles could not go under the shelf in group pens. Raw data are presented with analyses conducted on transformed data grouped into 4 h time periods for locations of ‘in water’ **(A)**.

There was a significant interaction between pen type and time period [*F*_(5,4342)_ = 107.42, *p* < 0.0001] for the crocodiles in the water during the scan observations (Figure 3A). Crocodiles in the unitised pens were observed more often in the water than on the shelf. Crocodiles in the group pens spent similar time in the water or on the shelf (Figure 3A). The diurnal pattern in the unitised pens showed crocodiles were on the shelf the most across daytime hours with peak use from noon to 18:00 (Figure 3A). Across all observations in the unitised pens, crocodiles were observed in the water 90.5% of observations, whereas in group pens, crocodiles were observed in the water 49.15% of observations (Figure 3A). Crocodiles in the unitised pens were most often observed being fully under the shelf with crocodiles in the group pens (who were unable to go underneath) showing some positioning alongside the shelf (Figure 3B).

### 3.3 Activity of crocodiles

**Figure 4:**
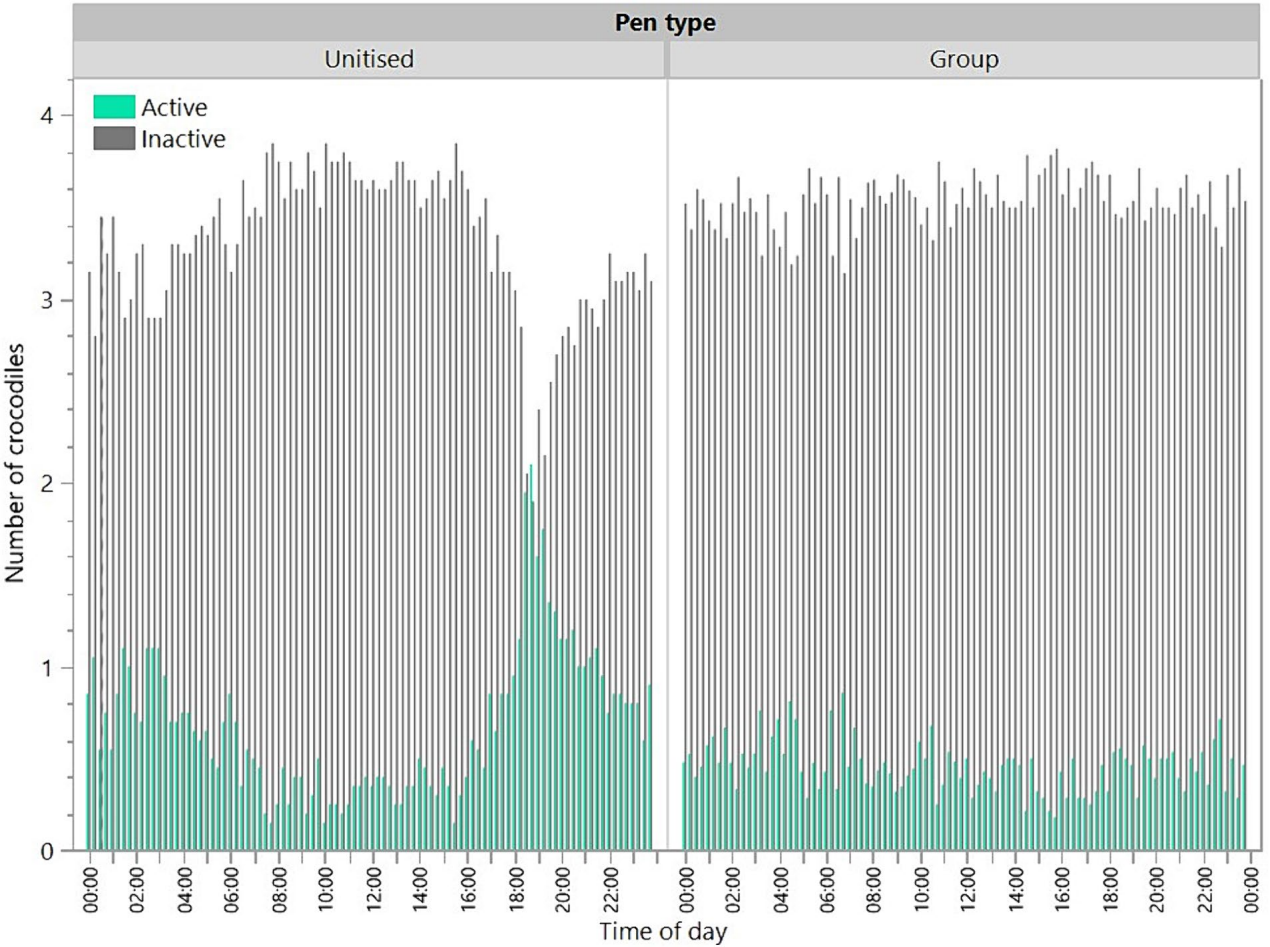
Number of crocodiles that were active or inactive at each 15 min scan observation point across the day for animals in unitised or group pens. Raw data are presented with analyses conducted on transformed data grouped into 4 h time periods.

There was a significant interaction between pen type and time period [*F*_(5,4342)_ = 20.31, *p* < 0.0001] for activity during the scan observations (Figure 4). Across both pen types, crocodiles spent less time active than inactive. The raw data presented in Figure 4 illustrate that the crocodiles in the group pens showed a consistent low level of activity throughout the day whereas the crocodiles in the unitised pens showed activity peaks in the late afternoon/evening (up to 2 of the 4 crocodiles observed active) with more activity from 16:00 through to 04:00 than the 12 h daylight period (04:00 through to 16:00).

### 3.4 Postures and behaviours of crocodiles

Numerical comparisons showed when in the water, crocodiles in the unitised pens had a variable pattern across time of being completely submerged with peaks throughout the night (Table 3). Comparatively, the pattern across time in the group pens was more evenly distributed (Table 3). Similarly, more variable patterns across time were observed for ‘nostrils and eyes out’ in the unitised pens relative to the group pens (Table 3). Observations of crocodiles with just their nose out varied across time in both pen types with different periods of peak time for their posture (Table 3).

**Table 3:** Mean (±SEM) number of crocodiles observed in five different postures while in the water across six time periods (across 24 h) while housed in unitised or group pens.

		Time period					
Pen type	Posture	00:15–04:00	04:15–08:00	08:15–12:00	12:15–16:00	16:15–20:00	20:15–00:00
Unitised	Submerged	2.20 ± 0.05	1.48 ± 0.07	0.45 ± 0.04	0.52 ± 0.04	1.42 ± 0.06	2.26 ± 0.05
	Nostrils and eyes out	0.22 ± 0.03	0.11 ± 0.02	0.08 ± 0.02	0.15 ± 0.02	0.46 ± 0.04	0.23 ± 0.03
	Head out	0.08 ± 0.02	0.03 ± 0.01	0.02 ± 0.007	0.04 ± 0.01	0.19 ± 0.03	0.11 ± 0.02
	Nose out	0.26 ± 0.027	0.13 ± 0.02	0.04 ± 0.01	0.04 ± 0.01	0.21 ± 0.03	0.29 ± 0.03
	Head and tail raised	0	0	0	0	0	0
Group	Submerged	0.89 ± 0.05	0.96 ± 0.04	0.94 ± 0.04	0.75 ± 0.04	0.64 ± 0.03	0.74 ± 0.04
	Nostrils and eyes out	0.19 ± 0.02	0.24 ± 0.02	0.23 ± 0.03	0.25 ± 0.02	0.23 ± 0.02	0.14 ± 0.02
	Head out	0.81 ± 0.04	0.92 ± 0.04	1.0 ± 0.05	0.76 ± 0.04	0.78 ± 0.04	0.89 ± 0.04
	Nose out	0.08 ± 0.02	0.13 ± 0.02	0.15 ± 0.02	0.16 ± 0.02	0.14 ± 0.02	0.09 ± 0.02
	Head and tail raised	0.003 ± 0.003	0.009 ± 0.005	0.02 ± 0.008	0.009 ± 0.005	0.004 ± 0.003	0.004 ± 0.004

Raw data are displayed.

Numerically, crocodiles in the group pens were more likely to be in the water with their head out than those in the unitised pens; while few individuals were observed with their head and tail raised in the group pens (*n* = 20 observations), none were observed in this posture in the unitised pens (Table 3).

**Figure 5:**
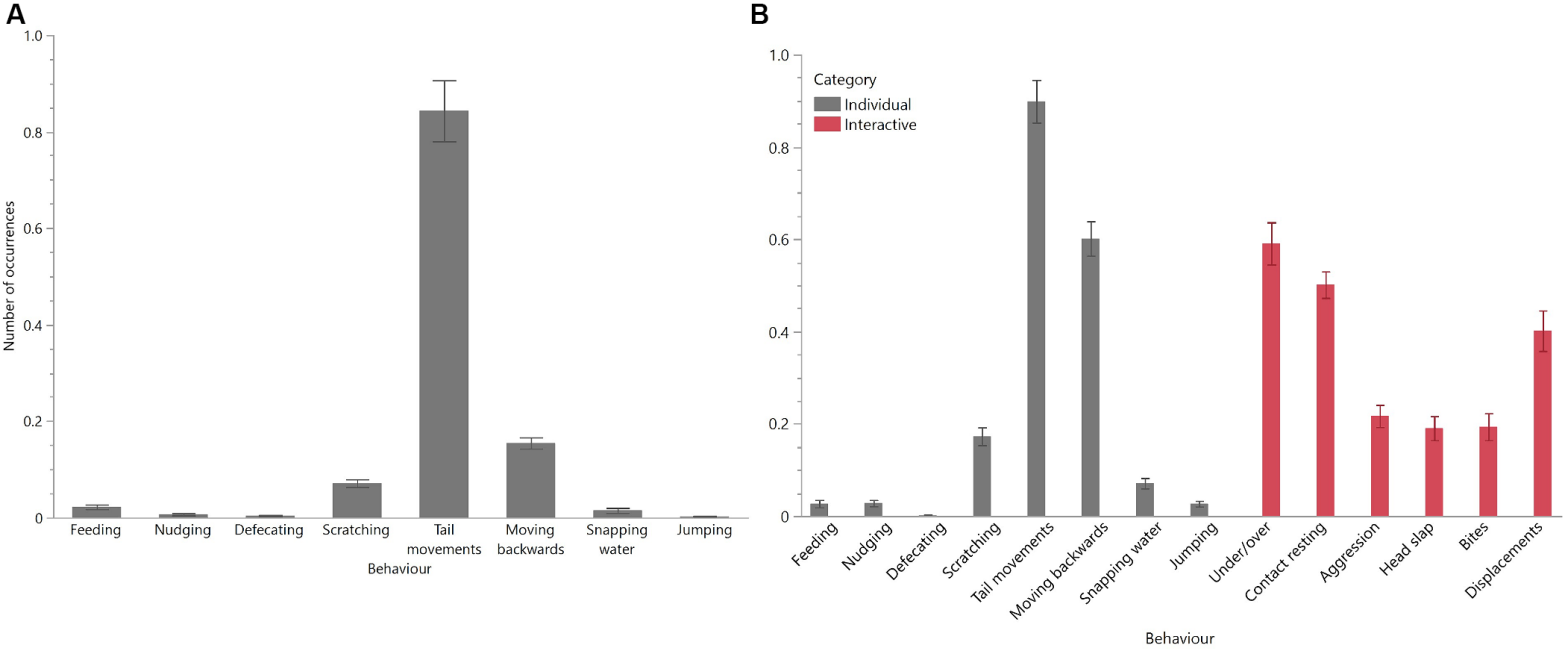
Mean (±SEM) occurrences of each behaviour for crocodiles housed in unitised **(A)** or group **(B)** pens. Crocodiles in the group pens could interact with each other and exhibited both ‘individual’ and ‘interactive’ behaviours **(B)**. Behaviours are described in the ethograms presented in Table 1. Raw data are displayed.

As expected, behavioural profiles were different between unitised and group pens with both socially interactive and aggressive behaviours observed in the group pens (Figures 5A,B). Overall, the most common behaviours observed in both unitised and group pens for individual crocodiles were tail movements, scratching, and moving backwards. In the group pens, individuals were observed socially interacting by climbing over and under each other and resting in close contact, but they were also observed exhibiting aggression towards each other (Figure 5B).

### 3.5 Free choice phase

**Figure 6:**
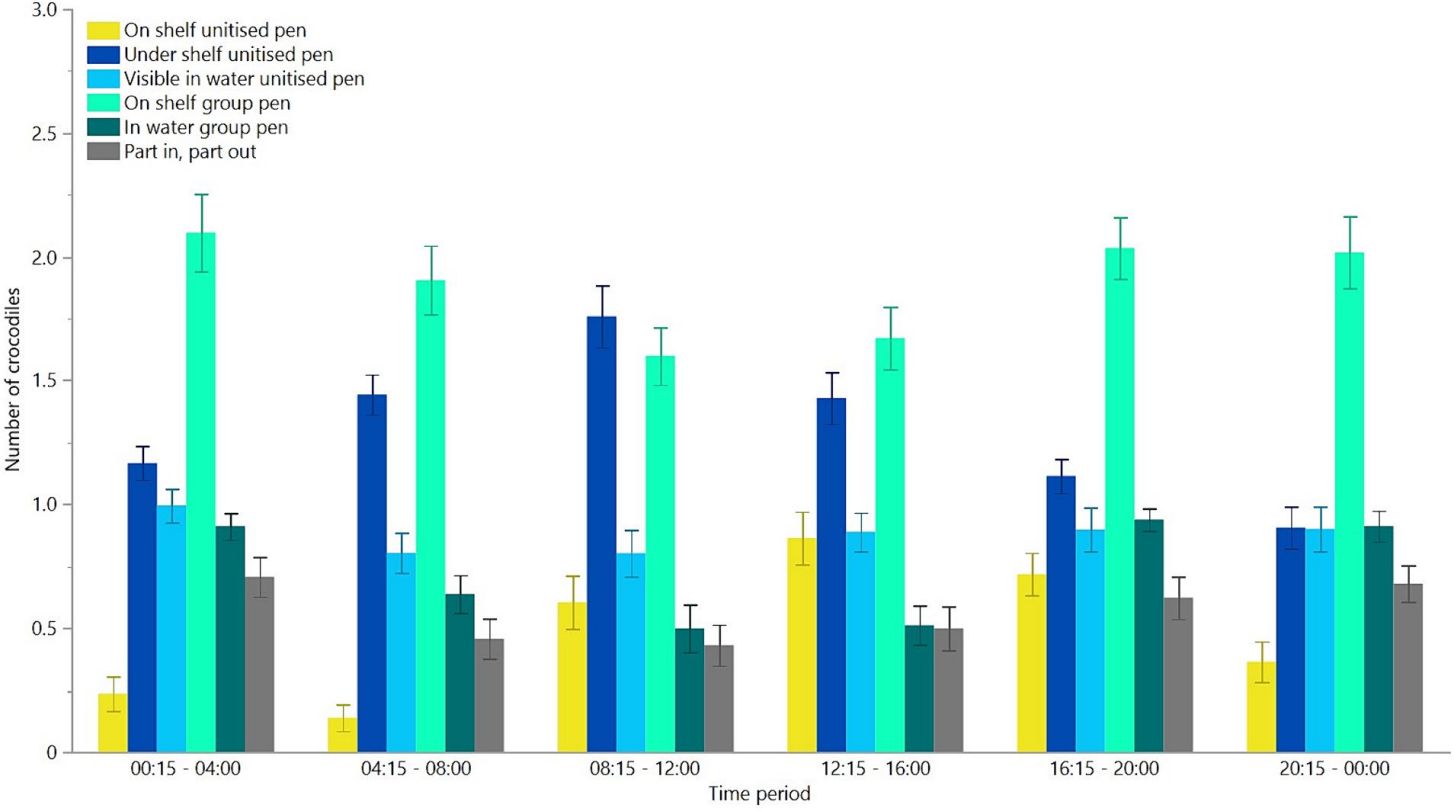
Mean (±SEM) number of crocodiles observed in six locations across six time periods throughout 24 h during free choice testing between group and unitised pens. It was not possible to go under the shelf in the group pen. Raw data are presented with analyses conducted on transformed values.

When given a choice between the unitised and group pens, there were significant differences in where the crocodiles preferred to be located and this varied among time periods across the day [*F*(_25,1408_) = 5.24, *p* < 0.0001, Figure 6]. Overall, there was also a significant effect of location [*F*(_5,1408_) = 66.28, *p* < 0.0001, Figure 6]. *Post-hoc* assessment of the differences in location showed that the crocodiles preferred to be on the shelf in the group pen or under the shelf in the unitised pens more than they were visible in the water in the group or single pen, or part in and part out between unitised and group pens, with the least preference for the shelf in the unitised pens (Figure 6).

### 3.6 Threat perception test

To initially determine an appropriate threat stimulus, four animals each were utilised to test three potential stimuli with the reactions monitored by both live and video observation. There were varying reactions observed between stimuli and between individuals (see Supplementary Tables S1–S3) with some subtle head/jaw movements only visible during the live observations due to shelf obstruction in the video recordings. The stimulus of ‘pressure on the thigh muscle of the hind limb with a broom handle’ elicited the strongest reactions (Supplementary Table S2) and thus was used in subsequent testing of the unitised pens, and group pens when a visible ‘cap wave’ elicited no response.

During administration of pressure for each threat perception test, the reactions of the individual crocodiles were varied for those reactions that could be seen from the video recordings with less variation observed in reactions of crocodiles in the group pens (Table 4). In the unitised pens, the majority of crocodiles were located under the shelf at the scheduled test time (11 of 12 test occurrences). Consequently, 26 of 47 observations were classified as ‘not visible,’ although they may have been able to be observed live by the farm personnel administering the test. There were eight instances of ‘no reaction’ observed in the group pens, but all crocodiles that were able to be observed in the unitised pens showed a reaction. In the group pens, 5 of 11 test occurrences the crocodile was on the shelf, and 6 of 11 the crocodile was in the water (only 11 tests were conducted as one crocodile was missed in replicate one). The variation in reactions was likely influenced by inconsistencies in the delivery of the pressure. It was unclear when there was no visible reaction or no strong reaction (e.g., lunge and snap) if this was because the individual was indifferent, or the pressure had been too gentle to elicit a reaction. Due to this confusion, there were some individuals that received more instances of pressure application than others (ranging from 1 to 10 applications per individual in the unitised pens and one to seven applications per individual in the group pens). Furthermore, subsequent observation of the video recordings showed that animals had pressure applied in varying body locations when in the group pen as it was logistically difficult to reach the hind leg in every animal. Pressure was applied to the left/right rear leg, by the tail, body, and belly. Consequently, these data were determined to be not reliable to inform a clear outcome of the threat perception test from a behavioural aspect. However, there was an effect on the faecal metabolome and microbiome, which will be reported on in a companion study.

**Table 4:** Behaviours observed per hind leg pressure application during the threat perception test of individual crocodiles in unitised and group pens.

Behaviour	Number of times observed	
	Unitised pens (*n* = 47 pressure applications)^1^	Group pens (*n* = 21 pressure applications)^2^
Not visible	26	
No reaction		8
Lunge snap	1	
Lunge no snap	1	
Tail movement	2	
Body moves away	5	5
Foot away	1	
Turn and snap	6	1
Turn	1	
Turn/lunges and snaps	4	7

1 Twelve individuals in unitised pens were tested across three replicates.

2 Eleven individuals in group pens were tested across three replicates.

### 3.7 Impact on body weight and skin quality

There were 5 female and 15 male animals used in the study. On entry to the test arena facility, they ranged from 1.55 to 1.89 m in length and 13.0 to 20.7 kg bodyweight (mean ± SD 16.75 ± 2.64 kg). As the animals had been pre-selected prior to the start of the first replicate, and replicates were sequential, each beginning at approximately 6-week intervals, the animals that were in the holding facility grew over the duration of the entire study. Thus, entry weights increased across the entire study, with the smallest animals in the first replicate and the largest in replicate 5. Within a replicate group of 4 animals, between two and four animals lost bodyweight with 15/20 animals losing body weight in total (range: 0.1–2.4 kg loss, mean ± SD 1.21 ± 0.67 kg loss). Body weight gain for the five animals (all male) ranged from 0.5 to 2.8 kg (mean ± SD 1.52 ± 0.91 kg gain). On entry to the test arena facility, skin scores ranged from 1 to 3 (mean ± SD 2.5 ± 0.76, a higher score indicates more damage), with the blemishes being old, healed scars, ‘pix’ (tiny full-thickness penetrating hole), and pitting. In terms of skin damage, with the exception of one animal that healed an existing blemish during the course of replicate 4 (this animal also gained weight), skin scores increased such that all scored 3, with fresh injuries noted on all animals. These injuries were described as ranging from ‘no major damage but several teeth marks’ to ‘multiple bite marks’ and ‘long scratch marks on the belly skin.’

## 4 Discussion

This study looked at the behaviour of grower saltwater crocodiles (in the finishing period) on a commercial farm under two types of pen housing systems, individual (unitised) or group, as well as preferences when they were given a choice between the two types. The results showed clear differences in where the animals spent their time across the day between the unitised and group pens as well as their activity levels in the different pens. However, interpretation of these differences is confounded by the physical and social differences between the pen types as crocodiles could access underneath a shelf in the unitised but not group pens. There were also differences in behaviours exhibited given there were social opportunities in the group pens where individuals were observed engaged in both aggressive and non-aggressive contact interactions. In the free choice environment, crocodiles spent similar amounts of time in both unitised and group pens, suggesting they did not always avoid social encounters. However, skins were damaged from teeth marks highlighting the physical and economical risks to group housing. Further work is needed to validate behavioural tests as possible future measures of affective state in farmed crocodilians.

The findings on crocodile location where more time was spent in the water in the unitised than group pens suggest that crocodiles prefer to spend time in the water. In the group pens, at any one observation, only two out of the four crocodiles in each replicate were in the water and the daily activity patterns were more uniform than in unitised pens. This may be a result of a dominance hierarchy, with the dominant animal(s) taking up residence in the water and preventing the more subordinate animals from accessing the water at times that they would prefer (1, 13). Subordinate animals could also have exhibited greater motionlessness to avoid aggression from dominant animals. The presence of a dominance hierarchy in the group housing situation was further supported by the weight loss/gain results where subordinate animals may have been prevented from feeding, similar to patterns seen in captive juvenile Nile crocodiles (30). The personnel caring for the animals did anecdotally observe a reduction in feed intake when the animals were moved from the unitised pens to the group pen. With reference to the air and water temperature data gathered, the location of the animals in the unitised pens suggests that crocodiles remained in the water overnight when the air temperature was lower than the water temperature, and they basked on the shelves between 10:00 and 20:00, with peaks at approximately 13:00 and 17:00 coinciding with peak air temperatures. These preferences for the water are likely related to crocodiles (as ectotherms) maintaining thermoregulation (1). There was a dip in basking behaviour at approximately 13:00–15:00, which may be associated with post-lunchbreak human activity on the farm. However, in the group pens, 50% of the crocodiles were on the shelf at any one time throughout the 24 h period. This suggests that preferred thermoregulation patterns may not be possible for some animals due to dominant animals preventing access. Animals that are on the shelf could become chilled at night when air temperatures drop and need to spend more time on the shelf during the day to warm up if they were unable to access the temperature-controlled water at night. This may result in increased stress in crocodiles that are unable to maintain optimal thermoregulation.

In the unitised pens, when crocodiles were in the water, there was a peak time period of being completely under the shelf from approximately 06:00 until 16:00, which coincided with daylight hours, as well as human activity on the farm. This could be related to the crocodiles seeking protection for long periods of submergence resting. Crocodilians of all ages have been observed to seek shelter in the wild, although particularly younger animals (7). There was no under-shelf area in the group pens to align with commercial grower pens and ensure visibility of all animals for monitoring purposes. In these pens, the crocodiles showed some positioning alongside the shelf at lower levels, which may have been indicative of motivation to seek cover if that option had been available. However, under the current study design, in which crocodiles in the group pen could not retreat under a shelf, it is not possible to separate the trade-offs between the motivation to seek shelter and the motivation for thermoregulation. Further work is required to understand these preferences and trade-offs.

Despite the possible restrictions on thermoregulation and/or inability to hide as well as a dominance hierarchy when in a group, during the free choice, crocodiles still chose to be in the group pen. In terms of behaviours exhibited in the group pens (not during free choice), the ‘head and tail raised’ posture may be a social behaviour related to aggression or a defence posture (6), as an attempt to appear bigger and therefore deter another crocodile from attack. The ‘in water with head out’ may be indicative of a level of vigilance in the group setting. However, they were also observed to be resting in contact with each other, as shown in other observations of younger captive *C. porosus* hatchlings (31). Thus, group settings may have both social consequences and benefits influencing the choice for crocodiles to be in close proximity rather than always separated, but more research is needed in this area. Aggressive interactions per crocodile could be considered to be relatively infrequent in number, but each interaction is a potential source of stress, and when one animal snaps at another, or clamps hold of a snout or limb with its jaws, there is the risk of injury, pain, and infection. Furthermore, biomechanically, when in slow motion, crocodiles tend to slide across a surface rather than raise up and walk across it. This means that when crocodiles slide over one another, the soft underbelly of the uppermost animal is exposed to injury (scratches or puncture) from the protruding lower teeth of the animal below. There needs to be greater understanding of the motivation for social contact to determine whether the social benefits of group housing provide enough positive impacts to outweigh any consequences from aggression and dominance.

The threat perception tests applied in this study were an attempt to quantify how the affective state of the crocodiles was impacted by the unitised versus group housing. Threat perception or attention bias tests have been validated and widely used across a range of species, including farmed livestock as a measure of anxiety [e.g., (32–34)], although application of similar behavioural tests in reptiles is limited (22). While the threat perception testing in the current study followed successful protocols used for other species, conducting these in the commercial housing environment presented multiple challenges. It was not feasible to move individuals to a specific testing arena as it is typical in other livestock species (32–34) and testing relied on farm rather than research personnel. Furthermore, validation of these tests to ensure they are reliably measuring affective state and refining the exact methods can take multiple iterations of testing (35). Thus, while the test results were unable to provide reliable information on crocodile affective state, they did highlight the need for further development of a rapid, practical, behavioural assessment test that is able to be consistently delivered in commercial conditions.

This study showed that behavioural profiles of commercial saltwater crocodiles differ when they are housed in unitised versus group pens, but it was not possible to disentangle the impacts of social restrictions from the lack of a sheltered under-shelf area in the group pens, nor the differences in pen sizes. The animals showed aggression when they were group-housed which resulted in bite marks that could compromise animal health as well as result in unsaleable skins but during free choice individual crocodiles still chose to be in contact with each other. The decision to spend time in both the unitised and group pens when presented with a choice suggests there are features of both pen types that are attractive to the crocodiles. These results can help inform management guidelines designed to optimise farmed crocodilian welfare. Further work could validate behavioural tests to quantify affective state impacts in different housing environments and whether social interactions do provide benefits for improving crocodile welfare.

## Data availability statement

The raw data supporting the conclusions of this article will be made available by the authors, without undue reservation.

## Ethics statement

The animal studies were approved by CSIRO Wildlife and Large Animal, Animal Ethics Committee (CWLA), reference 2020–20. The studies were conducted in accordance with the local legislation and institutional requirements. Written informed consent was obtained from the owners for the participation of their animals in this study.

## Author contributions

DC: Data curation, Formal analysis, Visualization, Writing – original draft, Writing – review & editing. LH: Conceptualization, Funding acquisition, Investigation, Methodology, Project administration, Writing – review & editing. CL: Conceptualization, Investigation, Methodology, Project administration, Resources, Supervision, Validation, Writing – review & editing. CT: Data curation, Investigation, Writing – review & editing. AS: Conceptualization, Data curation, Funding acquisition, Investigation, Methodology, Project administration, Resources, Supervision, Validation, Writing – review & editing.
