# Supplementary material for: Behaviours of farmed saltwater crocodiles (Crocodylus porosus) housed individually or in groups
Source: Front Vet Sci. 2024 Jul 8;11:1394198. doi: 10.3389/fvets.2024.1394198 (PMC11261483; doi:10.3389/fvets.2024.1394198)
Supplement: Supplementary file 1 [file Table_1.docx]

Supplementary Material

Behaviors of farmed saltwater crocodiles (*Crocodylus porosus*) housed individually or in groups

Dana L. M. Campbell^1^, Leisha Hewitt^2^, Caroline Lee^1^, Charlotte A. Timmerhues^3,^ and Alison H. Small^1*^

*** Correspondence:** Corresponding Author: alison.small@csiro.au

**Supplementary Tables 1-3**

Observations of individual saltwater crocodile behaviour during a threat perception pilot testing of three different stimuli. Observations were conducted live and later verified via video recordings.

Table S1: Stimulus 1 – object splashed into the water (animals hiding under shelf).

| **Pen number** | **Stimulus** | **Response** |
| --- | --- | --- |
| 55 | First splash | No movement |
|  | Second splash | No movement |
| 54 | First splash | No movement |
|  | Second splash | Withdrew limbs |
| 53 | First splash | Withdrew hind limb |
|  | Second splash | Withdrew fore limb |
| 52 | First splash | Opened jaws slightly, showing teeth. No postural change^1^ |
|  | Second splash | Lifted head^1^ |

^1^These reactions were only visible during the live observations as they were obscured by the shelf in the video recordings.

Table S2: Stimulus 2 – pressure applied to the thigh muscle of the hind limb with the rounded end of a broom handle.

| **Pen number** | **Response** |
| --- | --- |
| 60 | Spun around, out from under the shelf then retreated back under the shelf |
| 59 | Lunged and snapped at broom handle, turned around then returned under the shelf |
| 58 | Gentle response – moved forward and away from the broom handle, head pushing out from under the shelf. Jaws remained shut |
| 57 | No response, remained hidden under the shelf |

Table S3: Stimulus 3 – tapped on a hind foot with the rounded end of a broom handle.

| **Pen number** | **Response** |
| --- | --- |
| 50 | No response, remained hidden under the shelf |
| 49 | No response, remained hidden under the shelf |
| 48 | No response, remained hidden under the shelf |
| 47 | No response, remained hidden under the shelf |
